# The Studies on Structure and Stability of CaB_n_ Clusters

**DOI:** 10.3390/molecules24061011

**Published:** 2019-03-13

**Authors:** Peilin Han, Fengli Chai, Bolin Qiao, Chunhui Liu

**Affiliations:** School of Chemistry and Chemical Engineering, Xuchang University of China, No. 88 of Bayi Road, Xuchang 461000, China; 18838470326@163.com (F.C.); QBL1126@163.com (B.Q.)

**Keywords:** Density Functional Theory (DFT), CaB_n_ (n = 1–8) clusters, Geometric structure, Stability

## Abstract

Calcium-boron systems have excellent properties of hardness, strength, and chemical stability, and we studied a series of CaB_n_ clusters to investigate their structures and relative stability. The results showed the most stable structures of CaB_n_ clusters are not planar. The B atoms tend to get together and form the planar ring to stabilize the structure, and the Ca atoms are coordinated to the periphery of the formations. The average binding energy (*E*_b_), fragmentation energy (*E*_F_), second-order energy difference (Δ_2_*E*), adiabatic detachment energy (ADE), and adiabatic electron affinity (AEA) of the CaB_n_ clusters were calculated to investigate the relative stability and the ability of removing or obtaining an electron. As shown by the results, *E*_F_ and Δ_2_*E* values had obvious odd-even alteration as n increased, which indicated that the formations CaB_4_, CaB_6_, and CaB_8_ were more stable. The ADE values for CaB_n_ clusters with even values of n were higher than those with odd values of n, which indicated CaB_n_ clusters with even values of n had difficultly removing an electron. The AEA values of CaB_3_ and CaB_7_ were larger than the others, which meant CaB_3_ and CaB_7_ easily obtained an electron. These results provide a useful reference for understanding the formation mechanism and stability of the alkaline earth metal boride as well as guidance for synthesizing the CaB_n_ clusters.

## 1. Introduction

The boride compounds display excellent properties of hardness, strength, and chemical stability because of their structural characteristics. Due to the discovery of the superconductivity of MgB_2_ [1], the alkaline earth metal borides have been of great concern [1,2,3,4,5]. As an important member of the alkaline earth metal boride, the calcium boron systems are mentioned by many scientists [2,3,4,5,6]. Tian et al. [2] successfully synthesized CaB_4_ crystals under high-temperature high-pressure (HPHT) conditions and pointed out a potential synthesis method for the CaB_2_ crystal. Tian et al. [3] also prepared the CaB_6_ polycrystalline samples by solid phase sintering. At the same time, Zhang et al. [4,5] prepared the different composition CaB_6_ films by direct current (DC) magnetron sputtering. Due to the excellent properties of the alkaline earth metal boride systems, theoretical scientists have also shown interest in them [7,8,9,10,11]. Li et al. [7,8,9] studied the series of MB_5_^+^ (M = Be, Mg, Ca, Sr) and MB_6_ (M = Be, Mg, Ca, and Sr). Ju et al. [10] studied the geometric structures, stabilities, and electronic properties of ${\mathrm{MgB}}_{n}^{\pm m}$ (n = 1–7 and m = 0, 1). Last year, our group studied the structures and stabilities of BeB_n_^+^ (n = 1–8) clusters [11]. There have also been many theoretical works reported on the structures and stabilities of small-metal-atom-doped boron clusters [12,13,14,15,16,17,18,19,20]. However, until now, no systematic investigation for the CaB_n_ cluster had been performed to see the effect of the Ca atom doping on boron clusters. In this context, we investigated the stable configurations of the small clusters CaB_n_ (n = 1–8) formed by adding one Ca heteroatom into the corresponding B_n_ cluster. The stable configurations CaB_n_ were analyzed and compared with MgB_n_ clusters [10]. We also calculated the average binding energy (*E*_b_), fragmentation energy (*E*_F_), second-order energy difference (Δ_2_*E*), adiabatic detachment energy (ADE), and adiabatic electron affinity (AEA) in order to evaluate the stability and ability of CaB_n_ (n = 1–8) clusters to obtain and remove an electron.

## 2. Computational Details

In this paper, all calculations were performed with the Gaussian 03 [21] program package. The geometries of CaB_n_ clusters were fully optimized by using the B3LYP [22,23] method with 6-311+G(d) basis set and were characterized as energy minima by frequency calculations at the same level. The zero-point energies (ZPE) were also obtained at this level. In order to get more reliable electronic energy, the single point energy calculations for all the local minima were obtained at the MP2 [24]/6-311+G(d) level based on the geometry optimized at the B3LYP/6-311+G(d) level. All the lower-lying isomer energies were obtained at the MP2/6-311+G(d) level with zero point energy correction from the B3LYP/6-311+G(d) level. In each group of isomers, the reference energy was taken as that of the lowest geometry. The *E*_b_, *E*_F_, Δ_2_*E*, ADE, and AEA of the CaB_n_ clusters were calculated to investigate the relative stability and the ability of removing or obtaining an electron at the same level of theory. The following formulas were used:

$$\begin{array}{l} E_{b}(n)=[nE(B)+E(\mathrm{Ca})-E({\mathrm{CaB}}_{n})]/n+1\\ E_{F}(n)=E(B)+E({\mathrm{CaB}}_{n-1})-E({\mathrm{CaB}}_{n})\\ \Delta_{2}E(n)=E({\mathrm{CaB}}_{n+1})+E({\mathrm{CaB}}_{n-1})-2E({\mathrm{CaB}}_{n}) \end{array}$$

where *E*(B), *E*(CaB_n_), *E*(CaB_n+1_), and *E*(CaB_n-1_) are the energies of the most stable structures of B, CaB_n_, CaB_n+1_, and CaB_n-1_, respectively.

$$\begin{array}{l} \mathrm{ADE}=E({\mathrm{CaB}}_{n}{}^{+})-E({\mathrm{CaB}}_{n})\\ \mathrm{AEA}=E({\mathrm{CaB}}_{n})-E({\mathrm{CaB}}_{n}{}^{-}) \end{array}$$

where *E* is the energy of the optimized structures of CaB_n_^+^, CaB_n_^−^, and CaB_n_, each in its vibrational ground state.

## 3. Results and Discussions

### 3.1. Stable Geometric Structures

**Figure 1:**
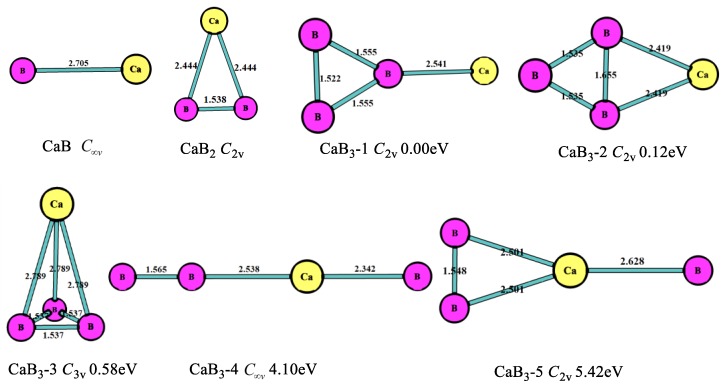
Geometry of the CaB_n_ (n = 1–3) clusters obtained at MP2/6-311+G(d)//B3LYP/6-311+G(d). The bond length unit is Å.

**Figure 2:**
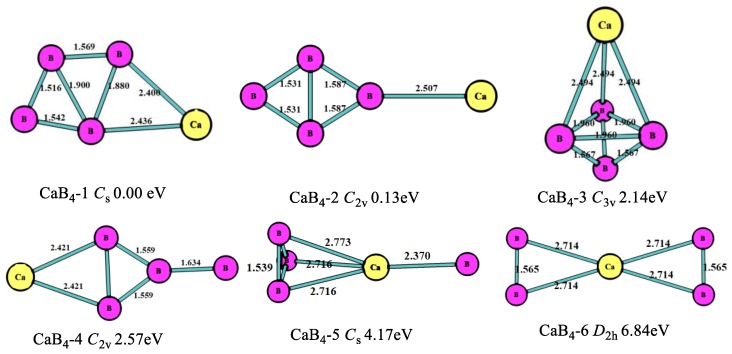
The geometry structures of the CaB_4_ clusters obtained at MP2/6-311+G(d)//B3LYP/6-311+G(d). The bond length unit is Å.

**Figure 3:**
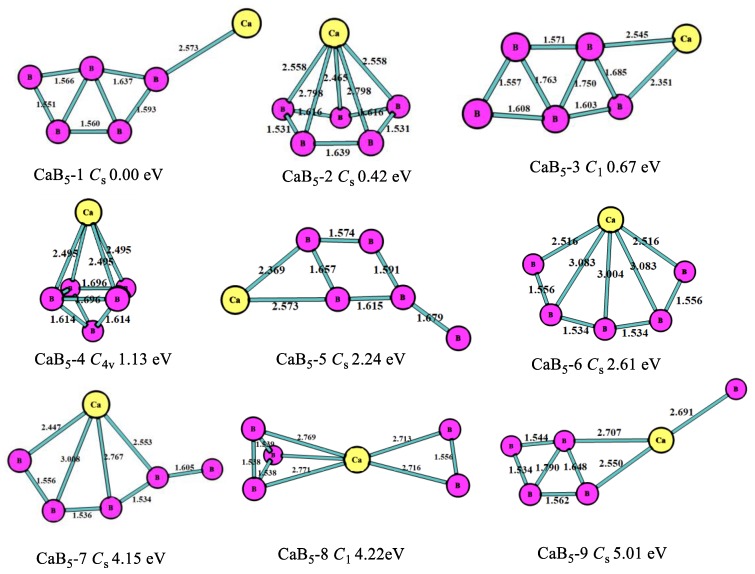
The geometry structures of the CaB_5_ clusters obtained at MP2/6-311+G(d)//B3LYP/6-311+G(d). The bond length unit is Å.

**Figure 4:**
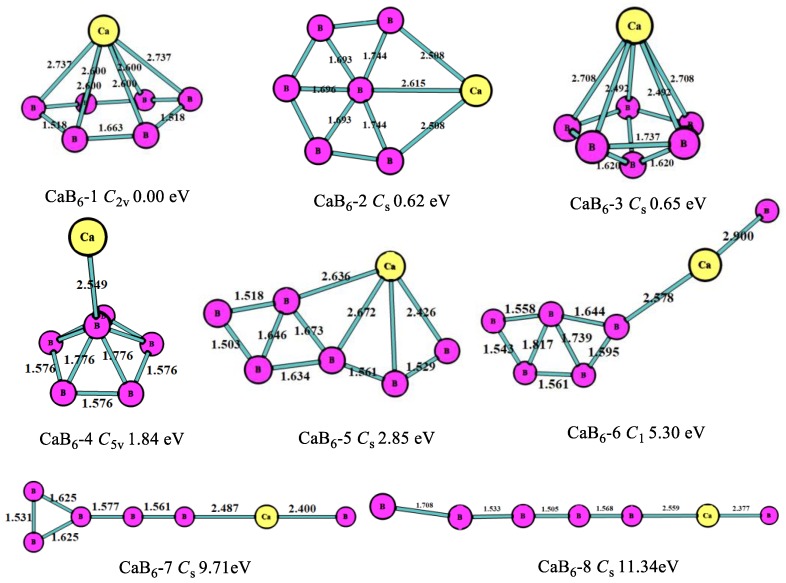
The geometry structures of the CaB_6_ clusters obtained MP2/6-311+G(d)//B3LYP/6-311+G(d). The bond length unit is Å.

**Figure 5:**
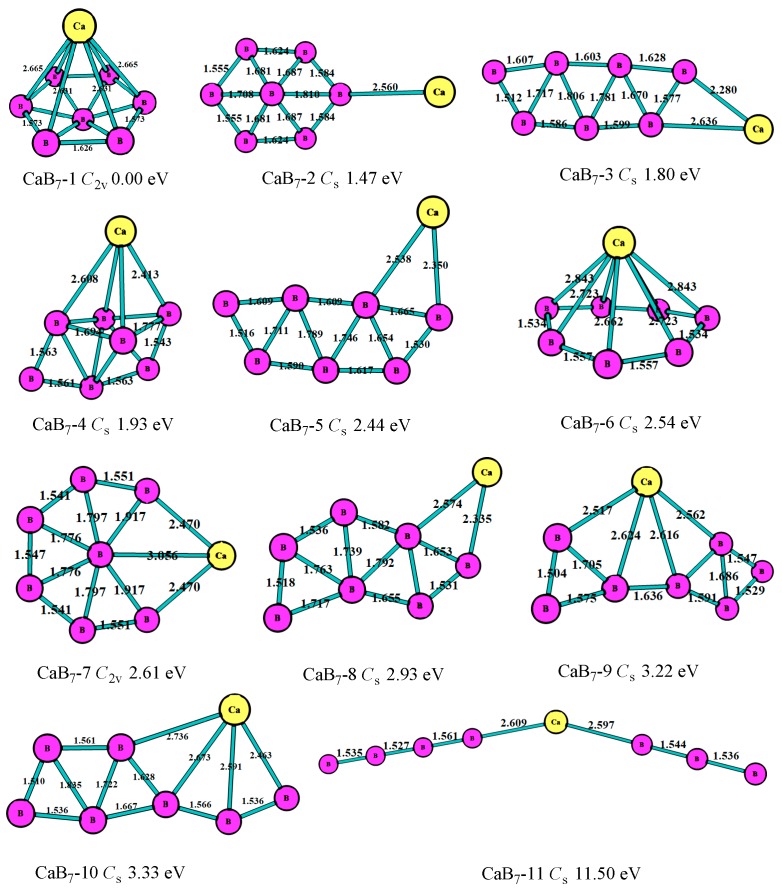
The geometry structures of the CaB_7_ clusters obtained at MP2/6-311+G(d)//B3LYP/6-311+G(d). The bond length unit is Å.

**Figure 6:**
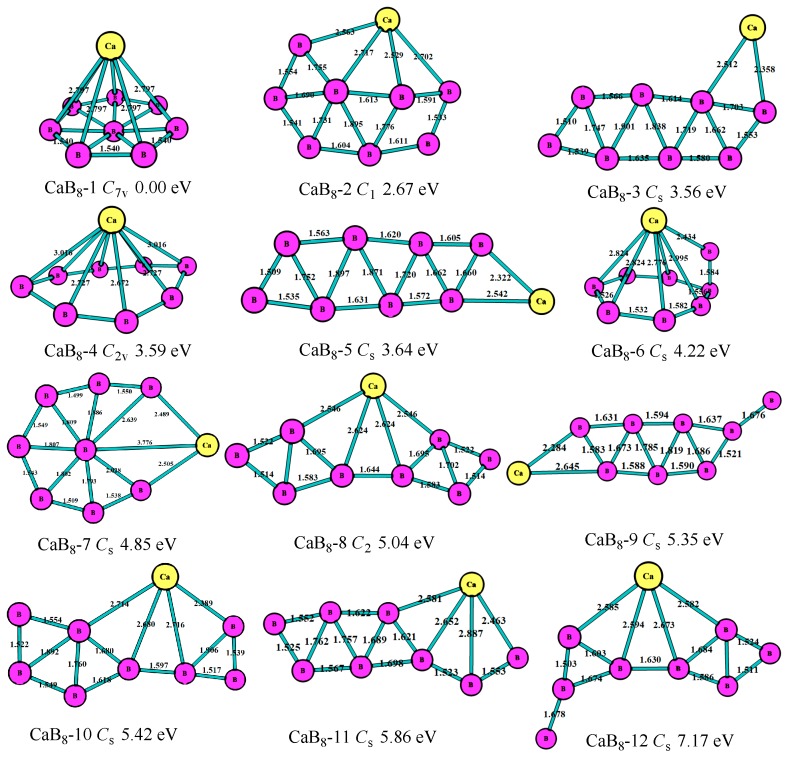
The geometry structures of the CaB_8_ clusters obtained at MP2/6-311+G(d)//B3LYP/6-311+G(d). The bond length unit is Å.

The optimized structures, symmetry point groups, and relative energies of the CaB_n_ (n = 1–8) clusters are displayed in Figure 1, Figure 2, Figure 3, Figure 4, Figure 5 and Figure 6 and Table 1. These structures included the lowest-energy structures and their low-lying isomers, and they were ordered from the lowest to highest energy. All of them had no imaginary frequency, and the relative energies were given in eV based on the most stable ones. All of the structures of CaB_n_ (n = 1–8) clusters were singlet state (where n is even number) or doublet state (where is odd number). 

As seen in Figure 1, for n = 1, the most stable structure was the linear geometry with the Ca-B bond length of 2.705 Å and point group $C_{\infty v}$. For n = 2, the most stable structure was triangle (CaB_2_, *C*_2v_), and none of the chain isomers of CaB_2_ were stable—that is, they were different from the MgB_2_ cluster structures [10]. The Ca-B bond length of CaB_2_ structure was 2.444 Å, remarkably longer than the Mg-B bond length of isomer MgB_2_ (2.265 Å) [10]. In the case of n = 3, the most stable geometry (CaB_3_-1, *C*_2v_) was the triangle boron ring with Ca connecting to one B atom in the B_3_ clusters, which was similar to the MgB_3_ cluster. The second stable one (CaB_3_-2, *C*_2v_) was the planar four-member ring including a triangle boron ring. The B-B bond length (1.655 Å) in CaB_3_-2 was longer than the B-B bond (1.555 Å and 1.522 Å) in CaB_3_-1, thus the energy of CaB_3_-2 was higher (0.12 eV) than that of CaB_3_-1. The third stable one (CaB_3_-3, *C*_3v_) was the trigonal pyramid structure with the Ca-B bond length of 2.789 Å. There was only a linear structure (CaB_3_-4) with the Ca atom at the middle of the chain. Above, the most stable structures of CaB_n_ (n = 1–3) were similar to MgB_n_ (n = 1–3).

As shown in Figure 2, the lowest-energy structure (CaB_4_-1, *C*_s_) was the planar five-member ring including a four-member boron ring, which was different from the MgB_4_ cluster. The second stable one (CaB_4_-2, *C*_2v_) was the four-member boron ring with the Ca connecting to one B atom in the B_4_ clusters, which had an energy higher (0.13 eV) than that of the most stable one. The third stable one (CaB_4_-3, *C*_3v_) was the tetrahedron, which was 2.14 eV higher than the most stable one. In this structure, the B-B bond lengths were 1.960 Å and 1.567 Å, and the Ca-B bond length was 2.494 Å. From the consideration of Figure 1 and Figure 2, we found B atoms tended to get together and make more stable structures. 

For n = 5, as shown in Figure 3, the most stable one (CaB_5_-1, *C*_s_) was the five-member boron ring with the Ca atom connected to the B atom of B_5_ clusters, which was similar to the most stable one of the MgB_5_ cluster [10]. The second lowest-energy structure (CaB_5_-2, *C*_s_) was the pentagonal pyramid including a five-member boron ring that was 0.42 eV higher in energy than CaB_5_-1 with *C*_s_ symmetry. The third most stable one (CaB_5_-3, *C*_1_) was the six-member ring including a five-member boron ring. The fourth lowest-energy structure was the quadrangular bipyramid with *C*_4v_ symmetry, which was 1.13 eV larger in energy than CaB_5_-1. As depicted in Figure 3, the B atoms of all the structures tended to get together and form the planar or quasi-planar boron clusters that were the same characters as in MgB_n_ and BeB_n_^+^ [10,11].

At n = 6, as shown in Figure 4, the lowest-energy structure (CaB_6_-1, *C*_2v_) for the CaB_6_ cluster was the hexagonal pyramid, which was different from MgB_6_ [10]. The second stable one (CaB_6_-2, *C*_s_) was the planar six-member ring with a B atom in the middle, which was similar to the most stable MgB_6_ structure. The third stable one (CaB_6_-3, *C*_s_) was the pentagonal bipyramid, which was 0.65 eV higher in energy than the most stable one. The fourth stable one (CaB_6_-4, *C*_5v_) was the pentagonal pyramid B_6_ with connected Ca atom. As is evident in Figure 4, the B atoms also tended to form planar or quasi-planar boron clusters and keep the structures more stable. This is because the planar or quasi-planar boron cluster stability is greatly defined by the aromaticity caused by the p- and d-delocalization [25], which requires a cyclic configuration consisting of at least three boron atoms. Thus, the clusters with cyclic boron configuration of CaB_n_ clusters are more stable than others. 

In the case of n = 7, as shown in Figure 5, the most stable (CaB_7_-1, *C*_2v_) of the CaB_7_ cluster was the hexagonal bipyramid geometry. The second lowest-energy structure (CaB_7_-2, *C*_s_) was the hexagonal pyramid of B_7_ with a connected Ca atom, which was similar to the most stable structure of MgB_7_. The third lowest-energy structure (CaB_7_-3, *C*_s_) was the planar seven-member ring, which was 1.80 eV higher than the most stable one. Figure 5 displays that most of the CaB_7_ clusters were not planar, which was different from that of the MgB_7_ clusters. As with the bond characteristics, the B atoms tended to get together and form the triangle boron ring, and the Ca atom was at the periphery. 

For n = 8, as shown in Figure 6, the lowest-energy structure (CaB_8_-1, *C*_s_) was the six-member ring of B_7_ with the Ca atom above the boron ring, which was the same with the most stable of the BeB_8_^+^ cluster [11]. The second lowest-energy structure (CaB_8_-2, *C*_2v_) was the seven-member ring with two B atoms above the ring, which had an energy 2.67 eV higher than that of the most stable one. The third lowest-energy structure (CaB_8_-3, *C*_s_) was the eight-member boron ring with the Ca atom connected, which was 3.56 eV higher than the most stable one. The fourth stable one (CaB_8_-4, *C*_2v_) was the eight-member boron ring with the Ca atom above the ring. The fifth stable one (CaB_8_-5, *C*_s_) was the nine-member boron ring with a Ca atom at the periphery. Figure 6 shows the B atoms also tended to get together to form the triangle boron ring to keep the structure more stable for the CaB_8_ clusters.

### 3.2. Relative Stability

**Figure 7:**
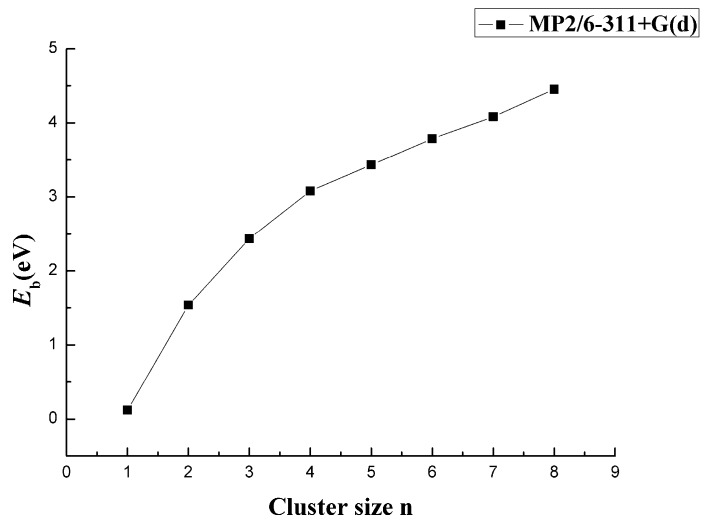
Size dependence of the average binding energy of CaB_n_ (n = 1–8) clusters.

**Figure 8:**
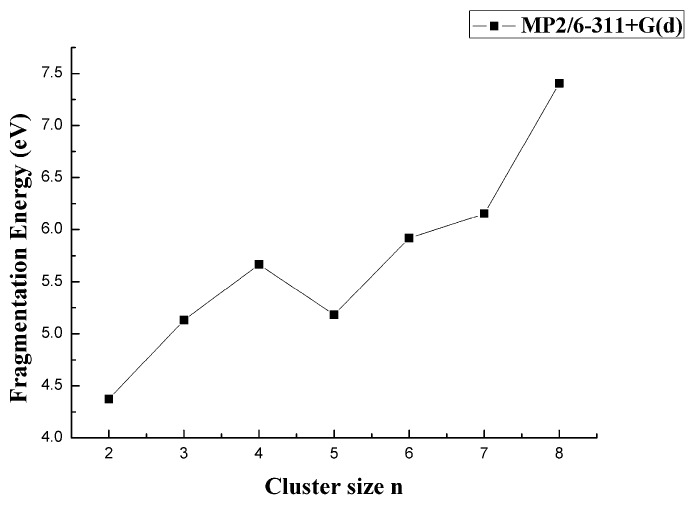
Size dependence of the fragmentation energy of CaB_n_ (n = 1–8) clusters.

**Figure 9:**
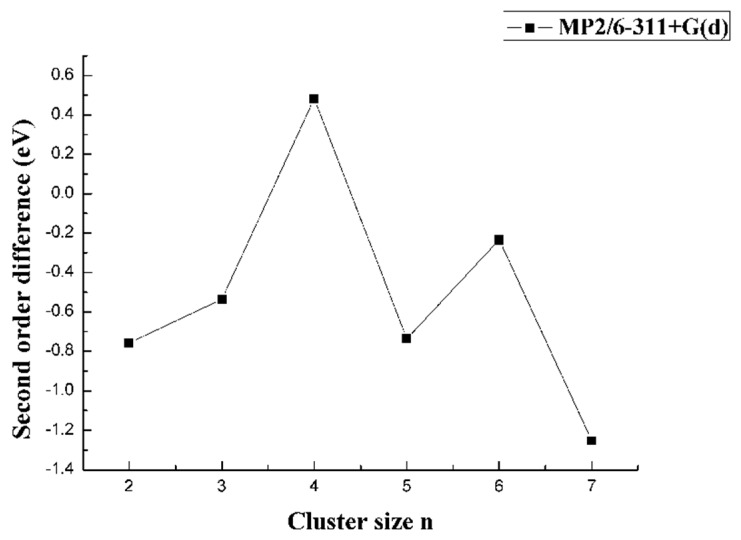
Size dependence of the second-order difference energy of CaB_n_ (n = 1–8) clusters.

To evaluate the relative stability of CaB_n_ clusters, Table 1 shows the *E*_b_, *E*_F_, and Δ_2_*E* at the MP2/6-311+G(d) level. These parameters were obtained by the following formula and plotted as the function of the cluster size n in Figure 7, Figure 8 and Figure 9.

In Figure 7, the energy *E*_b_ versus size n at the MP2/6-311+G(d) level is shown. All average binding energies *E*_b_ of the lowest-energy clusters increased as the size n increased, but after n = 3, the increase became smaller and stable. This was due to the bond tendency to saturate with the increase in the number of atoms. *E*_b_ increased as the number of B atoms increased. If the number of Ca atoms did not change, the system could form a stable, large-sized CaB_n_ cluster.

As shown in Figure 8 by the MP2 result for all the clusters, the global minimum of fragmentation energy appeared at n = 2. The energy followed obvious odd-even alterations as the size n increased from n = 3. However, it was noted that the fragmentation energy had its local-maximum when n was even, which indicated the CaB_4_, CaB_6_, and CaB_8_ were more stable. These results opposed those obtained for MgB_n_ and BeB_n_^+^. Among all the CaB_n_ (n = 1–8) clusters, the CaB_8_ cluster was the most stable.

The second-order difference in total energy is considered a very useful quantity that can reflect the relative cluster stability in the field of cluster physics [26]. From the MP2 result, the second-order difference energy followed a clear “odd-even oscillation” phenomenon for n = 3–7, as shown in Figure 9. When n was even, the value was at the peak, which indicated the higher stability of these clusters. The results were consistent with the information revealed in Figure 8. When n = 4, the second-order difference energy was the largest, which meant the CaB_4_ was the most stable among CaB_n_ (n = 3–7). 

### 3.3. The Ability of Obtaining or Removing an Electron

**Figure 10:**
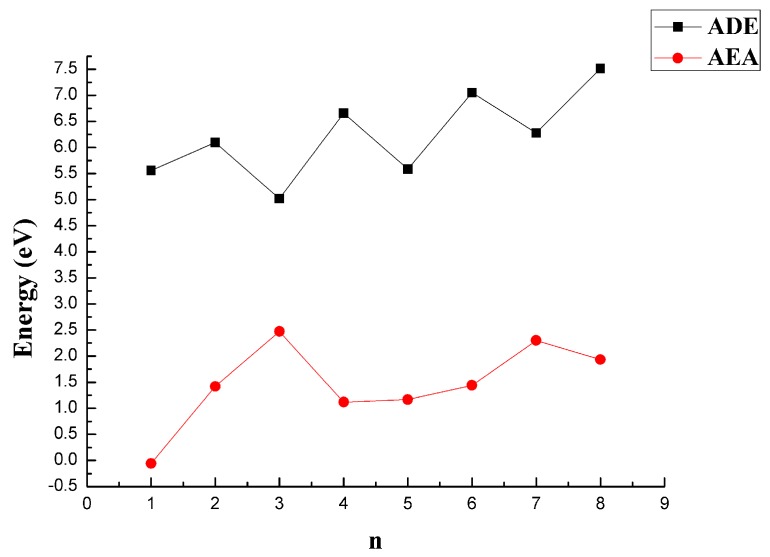
ADE and AEA of the most stable structures of CaB_n_ (n = 1–8) clusters at the MP2/6-311+G(d)//B3LYP/6-311+G(d) level.

The ADE and AEA of the CaB_n_ clusters were calculated to investigate the ability of the most stable CaB_n_ clusters to remove or obtain an electron. A larger value of ADE meant that it was difficult for the cluster to remove an electron, and a larger value of AEA meant that it was easy to gain an electron. The results are shown in Figure 10 and Table 1. The ADE values for CaB_n_ clusters with even values of n were higher than those with odd values of n. This indicated that CaB_n_ clusters with even values of n had more difficulty removing an electron than those with odd values of n. The AEA values first increased and then decreased over the range of n = 1–4, and they subsequently increased and then decreased for n = 4–8. The larger AEA values for n = 3 and n = 7 revealed that obtaining an electron was easy.

## 4. Conclusions

The geometries, stabilities, and electronic properties of CaB_n_ clusters up to n = 8 were systematically investigated using the B3LYP and MP2 method. It was found that the most stable structures of CaB_n_ clusters as n increased were not the planar configurations. The B atoms tended to form the planar ring to keep the structure more stable, and the Ca atoms were coordinated to the structure periphery. For the most stable structures, the average binding energy, the fragmentation energy, and second-order difference of total energies were widely used to evaluate the relative stability of clusters. The results showed they had obvious odd-even alterations as the size n increased. When n was even, it had its local-maximum, which indicated the CaB_4_, CaB_6,_ and CaB_8_ were more stable. The ADE and AEA of the CaB_n_ clusters were calculated to investigate the ability of removing or obtaining an electron. The results showed the ADE values for CaB_n_ clusters with even values of n were higher than those with odd values of n, which indicated CaB_n_ clusters with even values of n had difficultly removing an electron, and the AEA values of CaB_3_ and CaB_7_ were larger than the others, which meant CaB_3_ and CaB_7_ easily obtained an electron. 

## Figures and Tables

**Table 1:** The energies, the average binding energy (*E*_b_), the fragmentation energy (*E*_F_), second-order difference of total energy (Δ_2_*E*), adiabatic detachment energy (ADE), and adiabatic electron affinity (AEA) of the CaB_n_ clusters of the most stable structures of CaB_n_ at the MP2/6-311+G(d)//B3LYP/6-311+G(d) level.

CaB_n_	Energies/a.u.	*E*_b_/eV	*E*_F_/eV	Δ_2_*E*/eV	ADE/eV	AEA/eV
CaB	−701.5209	0.1196	—	—	5.5593	−0.0595
CaB_2_	−726.2515	1.5378	4.3741	−0.7567	6.0984	1.4198
CaB_3_	−751.0098	2.4361	5.1308	−0.5352	5.0175	2.4738
CaB_4_	−775.7879	3.0820	5.6660	0.4833	6.6577	1.1190
CaB_5_	−800.5481	3.4321	5.1826	−0.7357	5.5863	1.1684
CaB_6_	−825.3354	3.7873	5.9184	−0.2340	7.0507	1.4423
CaB_7_	−850.1314	4.0829	6.1523	−1.2530	6.2788	2.3017
CaB_8_	−874.9733	4.4521	7.4053	—	7.5131	1.9359
